# Inactivation of the Phosphatase Activity of Soluble Epoxide Hydrolase Modulates SIRT3 and Attenuates Experimental Pulmonary Hypertension

**DOI:** 10.1002/cph4.70108

**Published:** 2026-02-06

**Authors:** Matthieu Leuillier, Mustapha Chelgham, Hind Messaoudi, Ly Tu, Severine Ménoret, Raphaël Thuillet, Déborah Groussard, Felix F. Lillich, Mina Ottaviani, Lionel Nicol, Paul Mulder, Marc Humbert, Vincent Richard, Ignacio Anegon, Christophe Morisseau, Ewgenij Proschak, Christophe Guignabert, Jérémy Bellien

**Affiliations:** ^1^ INSERM U1096, EnVI Univ Rouen Normandie Rouen France; ^2^ INSERM UMR_S 999 Pulmonary Hypertension: Pathophysiology and Novel Therapies (HPPIT) Le Kremlin‐Bicêtre France; ^3^ Faculté de Médecine Université Paris‐Saclay, HPPIT Le Kremlin‐Bicêtre France; ^4^ CHU Nantes, Inserm, CNRS, SFR Santé, Inserm UMS 016, CNRS UMS 3556 Nantes Université Nantes France; ^5^ CHU Nantes, Inserm, Centre de Recherche en Transplantation et Immunologie, UMR 1064, ITUN Nantes Université Nantes France; ^6^ Transgenesis Rat ImmunoPhenomic Platform Nantes France; ^7^ Institute of Pharmaceutical Chemistry Goethe‐University Frankfurt am Main Germany; ^8^ Department of Pharmacology CHU Rouen Rouen France; ^9^ Department of Entomology and Nematology, and UCD Comprehensive Cancer Center University of California Davis California USA

**Keywords:** phosphatase activity, pulmonary hypertension, SIRT3, soluble epoxide hydrolase

## Abstract

**Introduction:**

Pulmonary hypertension (PH) is a severe cardiovascular disorder characterized by elevated pulmonary artery pressure caused by remodeling of the pulmonary circulation. This study aimed to investigate the role of the soluble epoxide hydrolase phosphatase domain (sEH‐P) in PH pathogenesis.

**Methods:**

The effects of sEH‐P genetic inactivation were evaluated in vivo using a CRISPR/Cas9‐mediated approach in two rat modes of PH: the monocrotaline and the Sugen/hypoxia model. To further explore the underlying mechanisms, complementary in vitro experiments were conducted in cultured human pulmonary artery smooth muscle cells (PA‐SMCs), where sEH expression was modulated.

**Results:**

sEH‐P inactivation attenuated experimental PH in both rat models, as demonstrated by reductions in mean pulmonary artery pressure and total pulmonary vascular resistance. Histological analysis showed decreased pulmonary artery muscularization and reduced collagen deposition in the right ventricle. Moreover, sEH‐P inactivation reduced sEH protein levels and enhanced SIRT3 expression in the lungs. Two‐hybrid interaction assays suggested that sEH indirectly regulates SIRT3 expression. In cultured human PA‐SMCs, altering sEH levels influenced SIRT3 expression, cell proliferation, and the levels of FoxO1, BCL2, and Bax proteins. In sEH‐P KI rat lungs, FoxO1 levels increased, while anti‐apoptotic BCL2 protein decreased.

**Conclusions:**

Our findings underscore the role of sEH‐P in the development and progression of PH, partly through its regulation of SIRT3 expression, cell proliferation, and apoptosis‐related proteins. Targeting sEH‐P emerges as a promising therapeutic strategy for PH.

## Graphical abstract

Genetic inactivation of the phosphatase activity of soluble epoxide hydrolase prevents the development of pulmonary hypertension in rats, partly through upregulation of SIRT3 expression and decreased cell proliferation and apoptosis‐related proteins. Targeting sEH‐P emerges as a promising therapeutic strategy for pulmonary hypertension.

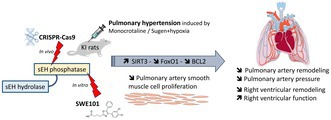

## 1 Introduction

Pulmonary hypertension (PH) is a progressive cardiovascular disorder characterized by elevated pulmonary artery pressure caused by an intense structural and functional remodeling of distal pulmonary arteries, ultimately leading to right heart failure and premature death (Simonneau et al. 2019; Humbert et al. 2023). Despite significant advancements in understanding PH pathogenesis, effective therapeutic strategies remain limited (Simonneau et al. 2019; Humbert et al. 2023). Therefore, there is a critical need to identify novel molecular targets and elucidate underlying cellular mechanisms to develop more effective interventions.

The mammalian soluble epoxide hydrolase (sEH) is a bifunctional enzyme consisting of a *C*‐terminal epoxide hydrolase activity (sEH‐H) and a more‐recently identified lipid phosphatase activity on its *N*‐terminal domain (sEH‐P), each potentially influencing PH development through distinct pathways (Morisseau and Hammock 2013). The sEH‐H catabolizes biologically active epoxides of polyunsaturated fatty acids, including epoxyeicosatrienoic acids (EETs), which are generated by cytochrome P450 enzymes (Morisseau and Hammock 2013; Kramer and Proschak 2017). Based on evidence that EETs constrict the pulmonary vasculature, sEH‐H inhibition was shown to potentiate hypoxic pulmonary vasoconstriction as well as chronic hypoxia‐induced pulmonary artery remodeling and PH in mice (Keserü et al. 2008, 2010). At the opposite, chronic inhibition of sEH‐H has not exacerbated PH in the Sugen‐hypoxia (SuHx) rat model and has even partially prevented PH development in the monocrotaline (MCT) rat model, likely due to its anti‐inflammatory effects (Leuillier, Platel, et al. 2023; Revermann et al. 2009).

Recent studies highlight the critical role of the mitochondrial deacetylase SIRT3 in cardiovascular health, including its protective function against PH by regulating mitochondrial bioenergetics and cell survival pathways (Cao et al. 2022; Paulin et al. 2014). FoxO1, a transcription factor regulated by SIRT3, modulates key cell‐cycle and apoptotic genes and has been implicated in controlling pulmonary artery smooth muscle cell (PA‐SMC) proliferation (Savai et al. 2014; Nie et al. 2024). The balance between anti‐apoptotic BCL‐2 and pro‐apoptotic Bax proteins further determines PA‐SMC survival and vascular remodeling dynamics (Chen et al. 2014). Previous reports suggest that sEH may influence these signaling axes (He et al. 2021), providing a rationale for investigating their interplay in PH pathogenesis.

In contrast, the biological function of the sEH‐P domain remains poorly understood. Using a unique transgenic rat model lacking sEH‐P activity, we recently demonstrated its role in the regulation of cardiometabolic regulation, partly through the metabolism of other intracellular lipid mediators including lysophosphatidic acids (Leuillier, Duflot, et al. 2023). Interestingly, differences have been observed between the effects of pharmacological sEH‐H inhibition and genetic knockdown of the entire sEH enzyme on pulmonary remodeling induced by chronic hypoxia in mice (Keserü et al. 2010). However, direct evidence of the involvement of sEH‐P remains lacking.

In this context, the present study aimed to evaluate the impact of sEH‐P genetic inactivation on PH in rat models and to explore the underlying mechanisms, with a particular focus on complementary experiments in human pulmonary artery smooth muscle cells (PA‐SMCs), which play a critical role in accumulating within the pulmonary arterial wall—a key event leading to lumen narrowing.

## 2 Methods

### 2.1 Animal Experiments

#### 2.1.1 Procedures

All animal care and procedures were approved by French Animal Experimentation Ethics Committees and conducted in accordance with the guidelines from the French National Research Council for the Care and Use of Laboratory Animals (Permit Numbers: Apafis #24107 and #11920). Experiments were performed in 10‐weeks‐old male wild‐type (WT) and sEH‐P knock‐in (KI) Sprague–Dawley rats bred at Janvier Labs (Le Genest‐Saint‐Isle, France). As previously described (Leuillier, Duflot, et al. 2023), the targeted gene mutation of the sEH gene (*EPHX2*) was achieved by substituting the catalytic nucleophile aspartic acid 9 with alanine (D9A) in the sEH‐P domain to abolish its phosphatase activity. This was accomplished using the clustered regularly interspaced short palindromic repeat (CRISPR)‐associated (Cas) DNA editing technology.

Rats were randomly assigned to three groups: a control group and two models of severe PH. WT and sEH‐P KI rats were subjected to either a three‐week treatment with MCT (40 mg/kg; Sigma‐Aldrich) or vehicle (saline). Another group received a single subcutaneous injection of Sugen 5416 (20 mg/kg) and was exposed to normobaric hypoxia for 3 weeks, followed by a 5‐week recovery period in room air (SuHx). At the end of the experimental period, echocardiography, cardiac MRI, and right ventricular (RV) hemodynamic measurements were conducted. Lung tissues were harvested for protein and histological analyses (Leuillier, Platel, et al. 2023).

#### 2.1.2 Cardiovascular Parameters

End‐diastolic and end‐systolic RV volumes, as well as ejection fraction, were assessed in methohexital‐anesthetized animals using a Bruker Biospec 4.7 Tesla MRI, and Cinema MRI images were acquired with the Integrate acquisition sequence (Bruker France, Wissembourg, France) (Leuillier, Platel, et al. 2023; Leuillier, Duflot, et al. 2023). RV hemodynamic measurements were performed in anesthetized rats (2% isoflurane in room air). A polyvinyl catheter was inserted into the right external jugular vein and advanced in the RV and pulmonary artery, allowing the measurement of right ventricular systolic pressure and mean pulmonary arterial pressure (mPAP) (Leuillier, Platel, et al. 2023). Cardiac output (CO) was determined by the thermodilution method, and total pulmonary vascular resistance (TPVR) was calculated as the ratio of mPAP to CO.

#### 2.1.3 Biological Analyses

##### 2.1.3.1 Histological Analysis

The left lung was removed and frozen for analysis, while the right lung was fixed in a distended state using formalin buffer. RV hypertrophy was evaluated by the Fulton index, and the percentage of muscularized pulmonary vessels was calculated. Immunohistochemistry and immunofluorescent staining were performed using antibodies against α‐smooth muscle cell actin (α‐SMA) and proliferating cell nuclear antigen (PCNA) (Dako, Les Ulis, France) as previously described (Leuillier, Platel, et al. 2023). In addition, the importance of collagen deposition in the RV was quantified using Picrosirius Red staining.

##### 2.1.3.2 Western‐Blot

Since sEH has been shown, independently of sEH‐H activity, to interact with and regulate sirtuin 3 (SIRT3) expression that plays a key role in PH development (Paulin et al. 2014; He et al. 2021), specific Western‐blot analyses were performed. Lung tissues were homogenized and sonicated in RIPA buffer supplemented with protease and phosphatase inhibitors. Protein samples (25–40 μg) were separated on SDS‐PAGE and transferred to a PVDF membrane. The membranes were incubated overnight at 4°C with primary antibodies to detect sEH, SIRT3, and downstream targets, including FoxO1, BCL2 (Savai et al. 2014; Nie et al. 2024; Chen et al. 2014), Bax, as well as β‐actin (Table S1). The BCL2/Bax ratio was calculated as the optical density of BCL2 band divided by that of Bax band, normalized to β‐actin. Chemiluminescence signals were detected using the Chemidoc Imaging System (Bio‐Rad), and Image Lab software was used to quantify the optical density of individual bands (Bio‐Rad Laboratories Inc). Protein expression was normalized to β‐actin.

##### 2.1.3.3 Confocal Microscopy Analysis

Immunofluorescence staining of α‐SMA, sEH, SIRT3, and FoxO1 was performed on paraffin‐embedded rat lung sections. At least three random lung sections (5‐μm thick) were deparaffinized and incubated in a retrieval buffer. The sections were then blocked with blocking buffer and incubated overnight with specific antibodies, followed by corresponding secondary fluorescently labeled antibodies (Thermofisher Scientific). Nuclei were stained with DAPI (Thermofisher Scientific). Slides were mounted using ProLong Gold antifade reagent (Thermo Fisher Scientific). Images were captured using an LSM700 confocal microscope (Zeiss) with ZEN software.

### 2.2 1‐by‐1 Yeast Two‐Hybrid System Interaction Assay

To assess a potential direct interaction between SIRT3 and sEH, 1‐by‐1 yeast two‐hybrid system interaction assays were performed with WT sEH or sEH‐P KI as bait proteins and SIRT3 as the prey protein (Hybrigenics Services SAS, France). The coding sequences of the bait proteins, WT sEH (GenBank accession number NM_022936.1) and sEH‐P KI (Morisseau and Hammock 2013), were PCR‐amplified and cloned in‐frame with the LexA DNA binding domain (DBD) into the plasmid pB27, which fuses the bait proteins to LexA at the C‐terminus (LexA‐bait fusion). pB27 is derived from the original pBTM116 vector. The coding sequence of the prey protein SIRT3 (GenBank accession number NM_001106313.2) was cloned in‐frame with the Gal4 activation domain (AD) into plasmid pP7 (AD‐prey fusion), derived from the original pGADGH. All constructs were verified by sequencing the inserts. Bait and prey constructs were then transformed in yeast haploid cells, L40deltaGal4 (matα) and YHGX13 (Y187 ade2‐101: loxP‐kanMX‐loxP, matα), respectively. Diploid yeast cells were generated using a mating protocol with both yeast strains (Fromont‐Racine et al. 1997). These assays were based on the HIS3 reporter gene, which is activated when the bait and prey interact (growth assay without histidine).

Negative controls included testing the bait plasmid with an empty prey vector (pP7) and testing the prey plasmid with an empty bait vector (pB27). The interaction between SMAD and SMURF was used as a positive control. Controls and interactions were assessed by streaking three independent yeast clones onto DO‐2 and DO‐3 selective media. The DO‐2 selective medium, which lacks tryptophan and leucine, was used as a growth control to confirm the presence of the bait and prey plasmids. The DO‐3 selective medium, which lacks tryptophan, leucine, and histidine, was used to select for interactions between the bait and the prey proteins.

### 2.3 Culture and Analysis of Primary Human Pulmonary Artery Smooth Muscle Cells (PA‐SMCs)

This study was approved by the local ethics committee (CPP Est‐III: N°ID RCB: 2018‐A01252‐53, N° CPP: 18.06.06), and all patients provided informed consent prior to participation. Lung specimens were obtained during lobectomy or pneumonectomy for localized lung cancer, collected from area distant from the tumor foci. Human PA‐SMCs were isolated and cultured as previously described (Leuillier, Platel, et al. 2023).

For proliferation assay, PA‐SMCs were seeded at a density of 5000 cells/well in a 96‐well plate and subjected to cell cycle arrest for 48 h in a medium lacking FBS with 0.1% BSA. Proliferation was then assessed by measuring 5‐bromo‐2‐deoxyuridine (BrdU) incorporation using a DELFIA kit (Perkin Elmer, Villebon‐sur‐Yvette, France), following the manufacturer's instructions. BrdU incorporation was quantified by measuring Eu‐fluorescence in a time‐resolved EnVision Multilabel Reader (PerkinElmer, Waltham, MA, USA).

For western‐blot analyses, PA‐SMCs were seeded and cultured during 48 h in medium with 10% FBS following the same protocol as described above.

Inhibition of sEH‐P was obtained by adding the sEH‐P inhibitor SWE101 (1 μM, synthesized by F.F.L. and E.P.) to the culture medium (Kramer et al. 2019). To knock down sEH expression, PA‐SMCs were transfected with scrambled RNA (106202318, Thermofisher Scientific) as a control or siRNA against sEH (10620319, Thermofisher Scientific) using Lipofectamine RNAiMAX, following the manufacturer's recommendations. To enhance sEH expression, PA‐SMCs were transfected using Lipofectamine 2000 with 1 ng of the sEH human construct in the pCMV6‐Entry Vector (p‐sEH, SC319259; OriGene, Rockville, MD, USA) or 1 ng of the pCMV6‐Entry mammalian expression vector (p‐Ctr, PS100020; OriGene). All experiments were performed 48 h after transfection.

### 2.4 Statistical Analysis

Data are presented as mean ± standard error of the mean (SEM). Statistical comparisons between two groups were performed using the non‐parametric unpaired Mann–Whitney test. For comparisons involving multiple groups, one‐way analysis of variance (ANOVA) followed by Tukey's post hoc test was applied. All statistical tests were two‐sided, and a *p*‐value < 0.05 was considered statistically significant. Statistical analyses were performed using GraphPad Prism software (version 10.2.3, GraphPad Software, San Diego, CA, USA).

## 3 Results

### 3.1 Inactivation of sEH‐P Activity Attenuates Experimental PH

**FIGURE 1:**
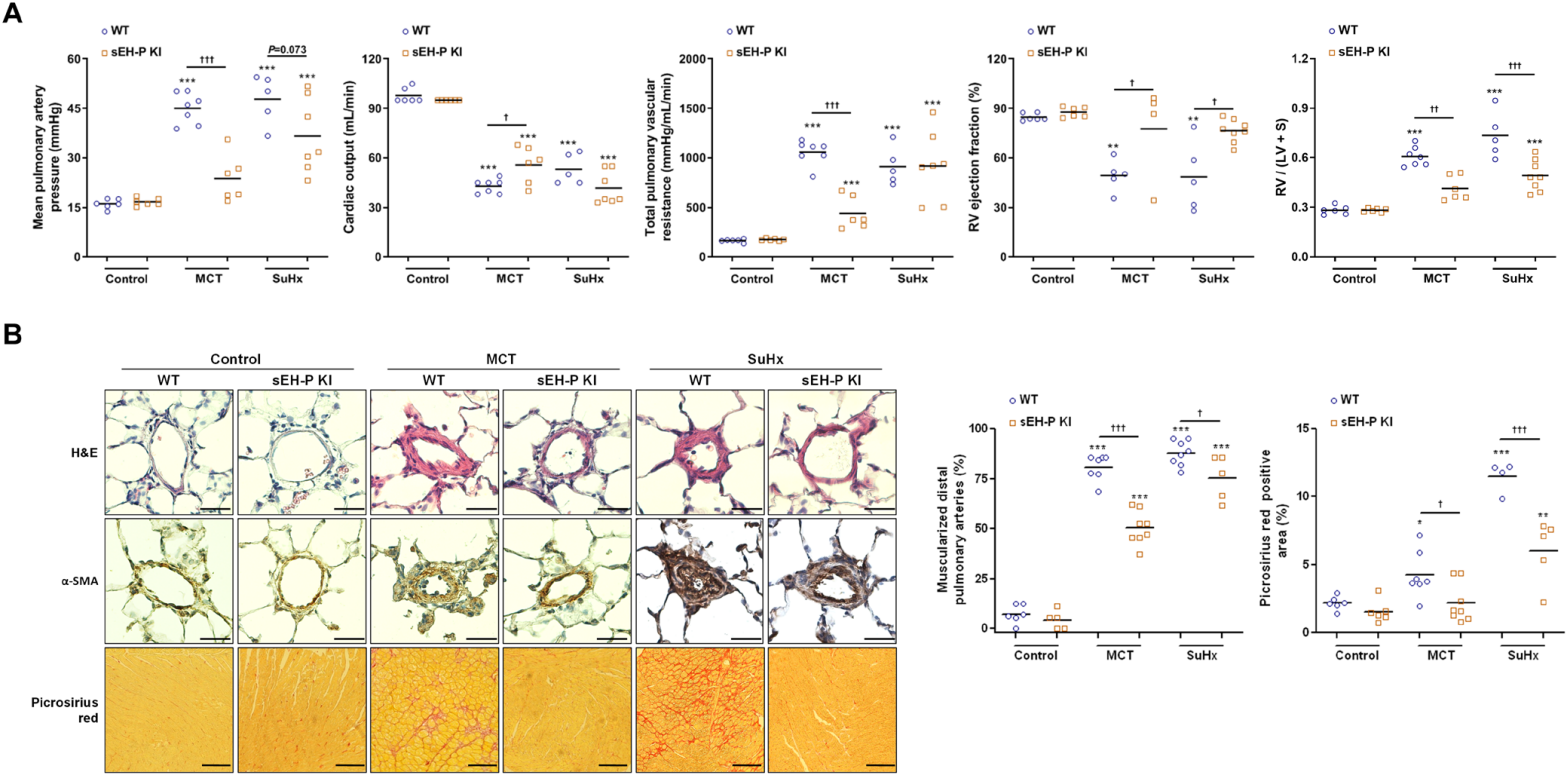
CRISPR/Cas9‐mediated inactivation of sEH‐P activity attenuates experimental pulmonary hypertension. (A) Measurements of mean artery blood pressure, cardiac output, total pulmonary vascular resistance, right ventricular (RV) ejection fraction, and Fulton index in WT and sEH‐P KI rats under control conditions and in the monocrotaline (MCT) and Sugen (SU5416) + Hypoxia (SuHx) models. (B) Histological analysis of lung sections using hematoxylin and eosin (H&E) staining, alpha‐smooth muscle Actin (α‐SMA) staining, evaluation of RV myocardium fibrosis based on Picrosirius Red staining, quantification of the percentage of muscularized pulmonary arteries, and quantification of the percentage of Picrosirius Red‐positive area in lungs from WT and sEH‐P KI rats under control conditions or in MCT and SuHx models. Scale bar = 50 μm. Data are presented as mean ± SEM with individual values (biological replicates). Statistical significance was determined using one‐way ANOVA with Tukey's post hoc tests. ***, *p* < 0.001 compared to the control condition. ^†^, *p* < 0.05; ^††^, *p* < 0.01; ^†††^, *p* < 0.001 compared to the corresponding WT condition.

The sEH‐P KI control rats exhibited normal mPAP, CO, TPVR, RV ejection fraction, and Fulton index compared to WT control rats (Figure 1A). Histological analysis of lung sections revealed no significant difference in the percentage of muscularized distal pulmonary arteries or in RV collagen deposition between sEH‐P KI and WT control rats (Figure 1B). As expected, in WT rats challenged with MCT and SuHx model, hemodynamic parameters worsened compared to WT controls, accompanied by increased muscularization of pulmonary arteries and RV collagen deposition. Interestingly, in rats with inactivated sEH‐P, the hemodynamic parameters, the percentage of muscularized pulmonary arteries, and collagen deposition in the RV return closer to normal values (Figure 1A,B).

Taken together, these results indicate that the CRISPR/Cas9‐mediated inactivation of sEH‐P activity significantly attenuated experimental PH, RV dysfunction, and vascular remodeling in MCT and SuHx‐challenged rats.

### 3.2 Inhibition of sEH‐P Reduces Proliferative Capacity In Vivo and In Vitro

**FIGURE 2:**
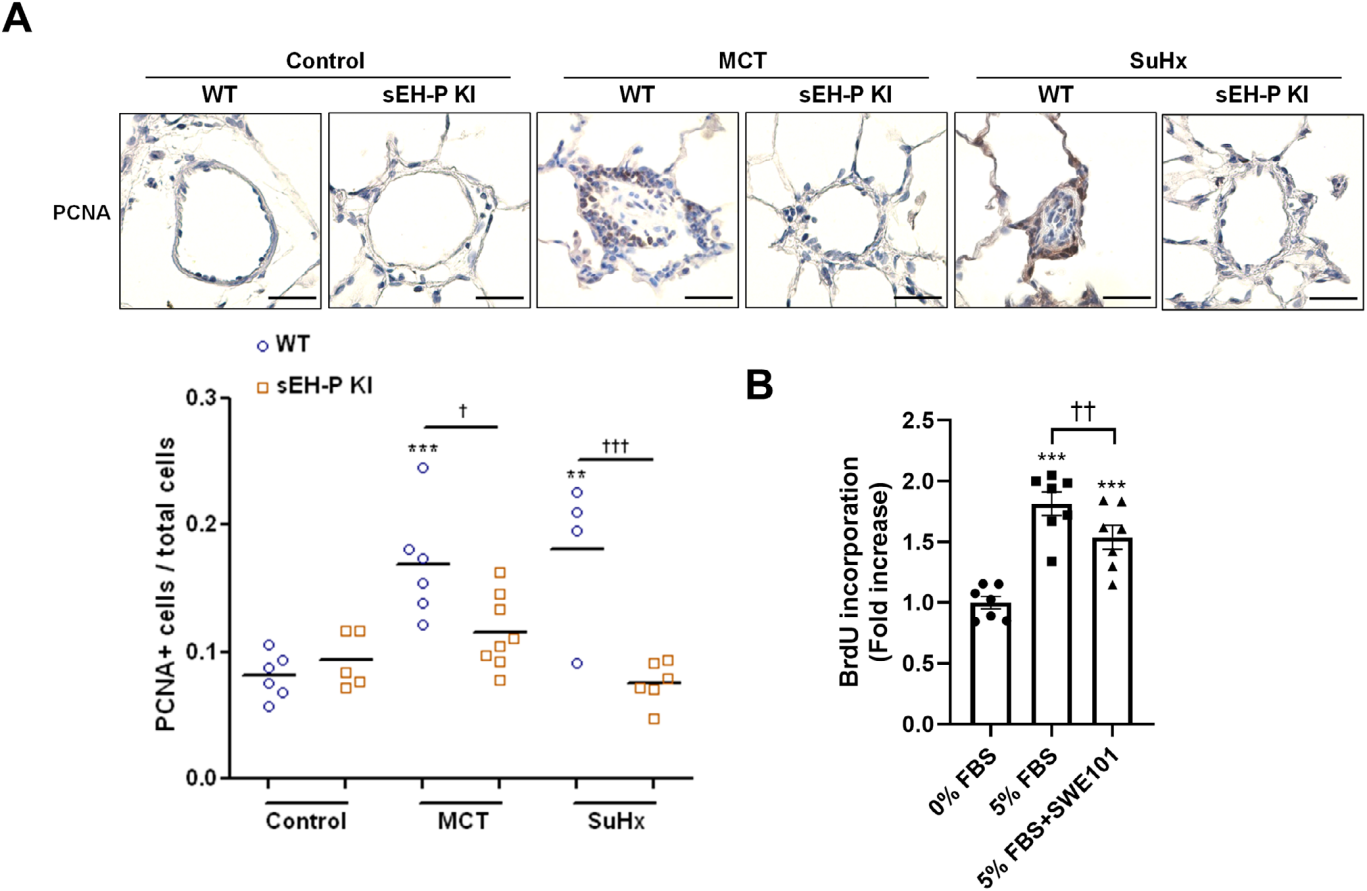
Inhibition of sEH‐P reduces proliferative capacity in vivo and in vitro. (A) Representative images and quantification of proliferation based on PCNA+ cells in WT and sEH‐P KI rats under control conditions and in the monocrotaline (MCT) and Sugen 5416/Hypoxia (SuHx) models. (B) Effect of the sEH‐P inhibitor SWE101 at 1 μM on the proliferation of pulmonary artery SMCs cultured in 0% or 5% FBS‐containing medium, measured by BrdU incorporation. Data are presented as mean ± SEM with individual values (biological replicates). Statistical significance was determined using one‐way ANOVA with Tukey's post hoc tests. **, *p* < 0.01; ***, *p* < 0.001 compared to control condition or 0% FBS. ^†^, *p* < 0.05; ^††^, *p* < 0.01 ^†††^, *p* < 0.001 versus WT or 5% FBS.

To explain the decreased muscularization observed in sEH‐P KI rats, the number of PCNA+ cells in the pulmonary arteries of WT and sEH‐P KI rats, either challenged or not with MCT or SuHx, was quantified. Under control conditions, no significant difference in PCNA+ cells was observed between WT and KI rats (Figure 2A). However, in both PH models, the pulmonary arteries of WT rats exhibited a 2‐fold increase in proliferating cells that was nearly normalized in sEH‐P KI rats (Figure 2A). These in vivo findings were confirmed in vitro, where human PA‐SMCs treated with the sEH‐P inhibitor SWE101 showed a significant reduction in proliferation compared to untreated cells (Figure 2B).

Collectively, these results indicate that inhibition of the sEH‐P activity leads to a reduction in the proliferative capacity of PA‐SMCs both in vivo and in vitro.

### 3.3 Inverse Correlation Between sEH and SIRT3 Protein Levels

To explore the molecular mechanisms underlying the protective effects of sEH‐P inhibition, Western blotting was performed on rat lungs with or without sEH‐P inactivation. Surprisingly, the results showed a significant reduction in sEH protein levels in the lungs of sEH‐P KI rats, a phenomenon previously not observed in metabolic organs (Leuillier, Duflot, et al. 2023). Notably, EPHX2 mRNA expression remained unchanged (Figure S1), indicating that this reduction occurs through a post‐transcriptional mechanism. Given the unchanged EPHX2 mRNA levels but reduced sEH protein abundance in sEH‐P KI lungs, altered protein stability represents a plausible mechanism. Future studies using cycloheximide chase assays to directly measure sEH protein turnover would be required to test this hypothesis.

**FIGURE 3:**
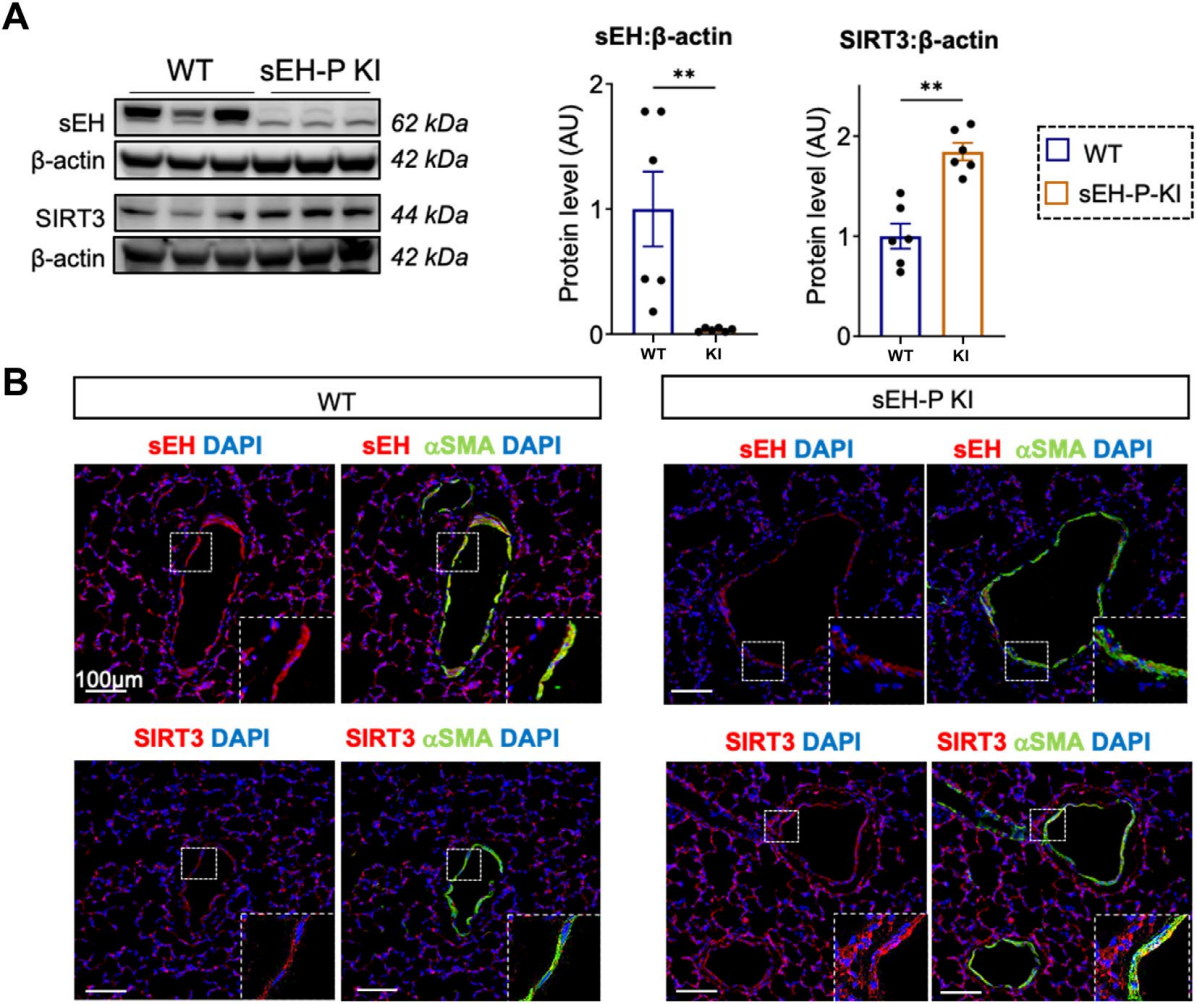
Inverse correlation between sEH and SIRT3 protein levels in sEH‐P KI rats. (A) Western blot analysis showing the pulmonary expression of sEH and SIRT3 in WT and sEH‐P KI rats. Data are presented as mean ± SEM with individual values (biological replicates). Statistical significance was determined using non‐parametric unpaired Mann–Whitney test. **, *p* < 0.01 versus WT. (B) Immunofluorescent staining of lung sections showing sEH, SIRT3 (red), α‐SMA (green), and nuclei (DAPI, blue) in WT and sEH‐P KI rats. Scale bar = 100 μm. Inset shows magnification of vascular wall.

As previously noted in the aortas of sEH knockout mice (He et al. 2021), this reduction in sEH was accompanied by an elevation of SIRT3 protein levels in the lungs of sEH‐P KI rats compared to WT rats (Figure 3A). Confocal microscopy analysis of lung sections allowed us to localize the decrease in sEH and increase in SIRT3 expression in the smooth muscle of the pulmonary arteries (Figure 3B). However, the yeast 1‐by‐1 interaction assays did not demonstrate a direct protein–protein interaction between sEH and SIRT3 (Figure S2), suggesting that sEH‐P KI likely increases SIRT3 expression through indirect effects. In vitro, the pharmacological inhibition of sEH‐P with SWE101 but also of sEH‐H with TPPU did not affect sEH nor SIRT3 protein expression levels (Figure S3).

**FIGURE 4:**
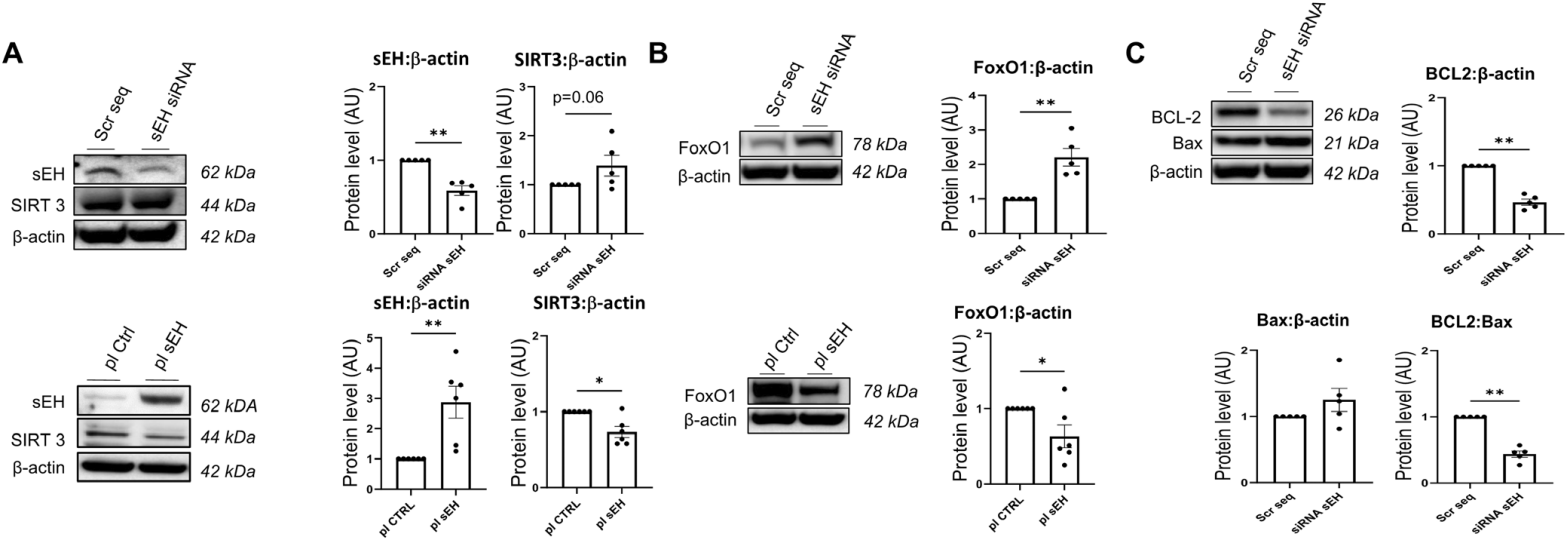
Downregulation of sEH expression level affects protein levels of SIRT3 and its downstream effectors FoxO1, BCL2, and Bax in cultured pulmonary artery SMCs. (A) Western blot analysis showing the protein expression of sEH and SIRT3 in pulmonary artery SMCs transfected with scrambled RNA or siRNA against sEH and with a control plasmid or a plasmid coding for sEH. (B) Western blot analysis showing the protein expression of FoxO1 in pulmonary artery SMCs transfected with scrambled RNA or siRNA against sEH and with a control plasmid or a plasmid coding for sEH. (C) Western blot analysis showing the protein expression of BCL2 and Bax in pulmonary artery SMCs transfected with scrambled RNA or siRNA against sEH. Data are presented as mean ± SEM with individual values (biological replicates). Statistical significance was determined using the non‐parametric unpaired Mann–Whitney test. *, *p* < 0.05; **, *p* < 0.01 versus control condition.

To investigate further, sEH protein levels were modulated in vitro. Knocking down sEH expression by 50% resulted in a modest increase in SIRT3 levels, whereas overexpressing sEH significantly decreased SIRT3 levels (Figure 4A). As observed in vivo, the reduction of sEH protein almost completely abolished the proliferation of PA‐SMCs induced by 5% FBS (Figure S4).

Collectively, these findings indicate that the loss of sEH, or its phosphatase domain, may lead to an up‐regulation of SIRT3 levels that contribute with unidentified mechanisms to the reduction in the proliferative capacity of PA‐SMCs both in vivo and in vitro.

### 3.4 Up‐Regulation of FoxO1 and Imbalance in BCL2/Bax Ratio

An increase in FoxO1 levels was observed in human PA‐SMCs with sEH knockdown, while decreased FoxO1 levels were noted when sEH was overexpressed (Figure 4B). Interestingly, sEH knockout PA‐SMCs also exhibited a reduced BCL2/Bax ratio (Figure 4C).

**FIGURE 5:**
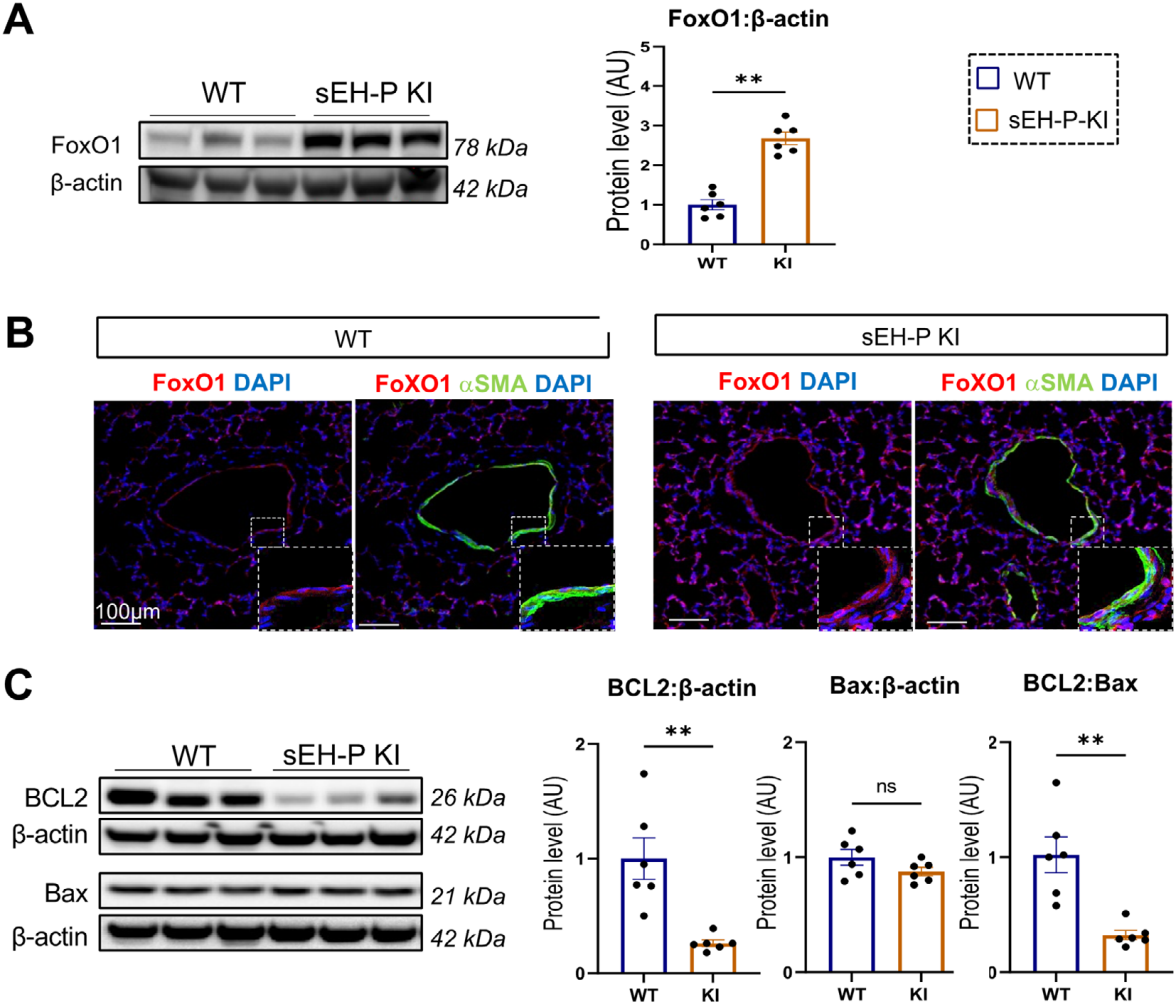
Increased levels of FoxO1 and decreased anti‐apoptotic BCL2 protein in sEH‐P KI rat lungs. (A) Western blot analysis showing the expression levels of FoxO1 in WT and sEH‐P KI rats. (B) Immunofluorescent staining of lung sections showing FoxO1 (red), α‐SMA (green), and nuclei (DAPI, blue) in WT and sEH‐P KI rats. (C) Western blot analysis representing the expression levels of BCL2 and Bax in WT and sEH‐P KI rats under control conditions and in the MCT and SuHx models. Scale bar = 100 μm. Inset shows magnification of vascular wall. Data are presented as mean ± SEM with individual values (biological replicates). Statistical significance was determined using non‐parametric unpaired Mann–Whitney test or one‐way ANOVA with Tukey's post hoc tests. **p < 0.01 versus WT.

Consistent with these in vitro observations, western blotting revealed an increased level of FoxO1 in the lungs of sEH‐P KI rats (Figure 5A). Immunofluorescent staining further confirmed that these changes were localized in the smooth muscle in the pulmonary arteries (Figure 5B). These same rats also displayed a reduction in the BCL2/Bax ratio (Figure 5C).

Taken altogether, these results suggest that sEH‐P KI rats are protected from the development of experimental PH through the up‐regulation of FoxO1 and an imbalance in the BCL2/Bax ratio. This imbalance may lead to reduced PA‐SMC proliferation and decreased resistance to apoptosis.

## 4 Discussion

The findings of this study underscore the contribution of sEH‐P in the pathogenesis of PH, establishing it as a promising therapeutic target. The CRISPR/Cas9‐mediated inactivation of sEH‐P activity in two well‐established rat models (MCT‐induced and SuHx‐induced PH) resulted in a substantial attenuation of disease features, including improved pulmonary hemodynamic parameters, reduced muscularization of pulmonary arteries, and decreased RV fibrosis.

At the molecular level, sEH‐P inactivation was associated with upregulation of SIRT3, a key regulator of mitochondrial function and cellular stress responses implicated in cardiovascular diseases (Cao et al. 2022). SIRT3 deficiency has been linked to mitochondrial dysfunction and exacerbation of PH, emphasizing the importance of its modulation in disease management (Paulin et al. 2014). While yeast two‐hybrid assays did not confirm a direct interaction between sEH and SIRT3 in pulmonary tissue, the findings suggest an indirect regulatory pathway potentially mediated by lipid phosphates metabolized by sEH‐P or its products (Morisseau and Hammock 2013; Kramer and Proschak 2017; Leuillier, Duflot, et al. 2023; Morisseau et al. 2012; Oguro and Imaoka 2012), providing novel insights into the complex biochemical interactions underlying PH pathogenesis. This interpretation is further supported by previous studies reporting that pharmacological inhibition of sEH‐H did not significantly affect PH progression in hypoxia‐exposed mice and rats (Keserü et al. 2010; Leuillier, Platel, et al. 2023). In contrast, in the present study, the pharmacological inhibition of sEH‐P with SWE101 reduced PA‐SMC proliferation, although it did not reproduce the decreased sEH expression or the upregulation of SIRT3 observed in vivo in the CRISPR sEH‐P knock‐in rats. These findings indicate that the antiproliferative effects of sEH‐P inhibition can occur independently of SIRT3 activation, while the in vivo genetic inactivation of sEH‐P may modulate mitochondrial and metabolic pathways, which could in turn contribute to the reduction in pulmonary pressure and improvement of RV function.

In addition to the upregulation of SIRT3, sEH‐P inactivation led to increased expression of FoxO1 and an altered BCL2/Bax ratio, favoring apoptosis over proliferation in PA‐SMCs. The interplay between these pathways is critical, as excessive PA‐SMC proliferation and resistance to apoptosis are hallmark features of PH (Li et al. 2024). FoxO1 has been shown to inhibit PA‐SMC proliferation by modulating key cell‐cycle regulators (Savai et al. 2014), and its upregulation in sEH‐P knock‐in rats further supports the role of this axis in attenuating vascular remodeling.

The in vitro experiments with human PA‐SMCs corroborated the in vivo findings, demonstrating reduced proliferation and altered expression of apoptosis‐related proteins upon inhibition of sEH‐P. These results provide translational relevance and suggest that pharmacological inhibition of sEH‐P could be explored as a novel therapeutic approach in humans. The potential to mitigate RV dysfunction and vascular remodeling via a single target represents a significant advancement in PH management, given the limited efficacy of current vasoreactive treatments.

Despite these promising findings, the study has limitations that warrant consideration. While the CRISPR/Cas9‐mediated sEH‐P inactivation model offers specificity, it does not fully replicate the potential off‐target effects or pharmacokinetics of pharmacological inhibitors in a clinical setting. At present, only a limited number of pharmacological sEH‐P inhibitors are available, and the existing compounds, including SWE101, display substantial off‐target effects and unfavorable pharmacokinetic properties that hinder their chronic in vivo use. Furthermore, this study primarily focused on male rats that showed a more pronounced cardiometabolic phenotype compared to females (Leuillier, Duflot, et al. 2023). This may not fully capture the sex‐specific differences in PH pathogenesis and response to treatment (Lahm et al. 2019). Furthermore, the indirect regulation of SIRT3 by sEH‐P remains incompletely understood, highlighting the need for further studies to identify the precise molecular intermediates and pathways involved. We cannot fully exclude that non‐enzymatic functions of sEH, such as protein scaffolding resulting from decreased sEH expression, may contribute to the beneficial effects observed in PH models. Lastly, the decreased muscularization of pulmonary arteries in sEH‐P KI rats may result from decreased cell migration in addition to the demonstrated reduction in cell proliferation.

In conclusion, this study underscores the contribution of sEH‐P in PH development and its potential as a novel therapeutic target. The ability of sEH‐P inactivation to reduce PA‐SMC proliferation, enhance apoptosis, and improve pulmonary hemodynamic and structural parameters in experimental models highlights its significance. Future research addressing the study's limitations, including development of specific and selective sEH‐P inhibitors, detailed molecular pathway investigations, and validation in diverse models, will be essential to advance sEH‐P‐based therapies for clinical application.

## Author Contributions

Ly Tu, EEwgenij Proschak, Christophe Morisseau, Christophe Guignabert, and Jérémy Bellien conceptualized the study. Mustapha Chelgham, Ly Tu, Matthieu Leuillier, Hind Messaoudi, Severine Ménoret, Mina Ottaviani, Ewgenij Proschak, Christophe Guignabert, and Jérémy Bellien designed and/or performed the experimental methods. Ly Tu, Vincent Richard, Christophe Guignabert and Jérémy Bellien contributed to project administration and supervision. Mustapha Chelgham, Hind Messaoudi, Christophe Guignabert, and Jérémy Bellien wrote the original manuscript draft, with input from all the authors. All authors reviewed and edited the manuscript.

## Funding

This study was supported by grants from the French National Research Agency (ANR‐16‐CE17‐0012, ANR‐22‐CE92‐0021, and ANR‐24‐CE17‐7692), the Deutsche Forschungsgemeinschaft (DFG‐505561502), the GCS G4 (FHU CArdiac Research Network on Aortic VALve and heart failure—CARNAVAL), the National Institute of Environmental Health Sciences (R35 ES030443 and P42 ES004699), European Union and Normandie Regional Council, and the Fondation pour la Recherche Médicale (FRM) grants no. EQU202203014670. Europe gets involved in Normandie with European Regional Development Fund (ERDF). M.L. and H.M. were recipients of Normandy Regional Council fellowship. In addition, the development of the sEH‐P KI rats was financially supported by the “TEFOR” project funded by the “Investissements d'Avenir” French Government program, managed by the French National Research Agency (ANRII INSB‐0014).

## Conflicts of Interest

Over the last 3 years, Christophe Guignabert reports grants from Acceleron, Structure Therapeutics, Corteria, and Diagonal Therapeutics and grants and personal fees from Merck, outside the submitted work. All other authors have no conflicts of interest to disclose.

## Supporting information


**Data S1:** cph470108‐sup‐0001‐Supinfo.doc.

## Data Availability

The data that support the findings of this study are available from the corresponding author upon reasonable request.
