# Supplementary material for: Inactivation of the Phosphatase Activity of Soluble Epoxide Hydrolase Modulates SIRT3 and Attenuates Experimental Pulmonary Hypertension
Source: Compr Physiol. 2026 Feb 6;16(1):e70108. doi: 10.1002/cph4.70108 (PMC12881838; doi:10.1002/cph4.70108)

**Inactivation of the Phosphatase Activity of Soluble Epoxide Hydrolase Modulates SIRT3 and Attenuates Experimental Pulmonary Hypertension**

Leuillier et al.

**Supplementary materials**

**Supplementary Table 1**

| **Antibody** | **Catalog number** | **Dilution** |
| --- | --- | --- |
| **sEH (human cell experiments)** | Provided by C.M. | 1:1000 |
| **sEH (rat experiments)** | PA5-102509, Invitrogen | 1:500 (WB) / 1: 100 (IF) |
| **SIRT3** | NBP3-02984, Novus Biologicals | 1:500 (WB) / 1: 100 (IF) |
| **FoxO1** | C29H4, Cell Signaling Technology | 1:500 (WB) / 1: 100 (IF) |
| **Bax** | Ab32503, abcam | 1:500 (WB) |
| **BCL2** | Sc-509, Santa Cruz Biotechnology | 1:200 |
| **α-SMA** | NB300-978, Novus Biologicals | 1:400 |
| **β-actin** | A3845, Sigma Aldrich | 1:25000 |

**Supplementary Figure S1. CRISPR/Cas9-mediated inactivation of sEH-P activity does not affect EPHX2 (sEH) mRNA expression levels.** RT-qPCR analysis showing the pulmonary mRNA expression of sEH in WT and sEH-P KI rats. Data are presented as mean ± SEM with individual values (biological replicates). Statistical significance was determined using non-parametric unpaired Mann-Whitney test.

**Supplementary Figure S2. Yeast two-hybrid assay showing no interaction between SIRT3 and sEH variants.** A 1-by-1 yeast two-hybrid system was used to assess direct interaction between SIRT3 (prey) and either WT sEH or sEH-P KI mutant as bait. Yeast diploids were streaked onto DO-2 selective medium (lacking tryptophan and leucine) to confirm the presence of bait and prey plasmids **(left panels)**, and onto DO-3 selective medium (lacking tryptophan, leucine, and histidine) to detect protein–protein interaction by growth without histidine **(right panels)**. **(1, 2)** WT sEH + SIRT3; **(3, 4)** sEH-P KI + SIRT3; **(5, 6)** positive control interaction (SMAD + SMURF). No growth observed on DO-3 indicates lack of interaction. Representative images show growth of three independent yeast clones for each combination.

**Supplementary Figure S3. Pharmacological inhibition of sEH-P does not affect sEH or SIRT3 protein expression *in vitro*.** Western blot analysis showing effect of sEH-H inhibitor TPPU (1 µM) and the sEH-P inhibitor SWE101 (1 µM) on sEH and SIRT3 protein expression in pulmonary artery SMCs. Data are presented as mean ± SEM with individual values (biological replicates). Statistical significance was determined using non-parametric unpaired Mann-Whitney test.

**Supplementary Figure S4.** Effect of sEH siRNA-mediated downregulation on the proliferation of pulmonary artery SMCs cultured in 0% or 5% FBS-containing medium, measured by BrdU incorporation (n=5). Data are presented as mean ± SEM with individual values (biological replicates). Statistical significance was determined using one-way ANOVA with Tukey's post hoc tests. *, p<0.05; ****, p<0.0001 versus scrambled sequence. $$, p<0.01 versus 0% FBS condition.


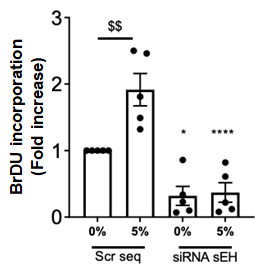

Supplement: Supplementary file 1 — Data S1: cph470108‐sup‐0001‐Supinfo.doc. [file CPH4-16-e70108-s001.doc]
